# Bridging Chronic Inflammation and Digestive Cancer: The Critical Role of Innate Lymphoid Cells in Tumor Microenvironments

**DOI:** 10.7150/ijbs.96338

**Published:** 2024-09-09

**Authors:** Guanliang Shen, Qi Wang, Zhengrui Li, Jiaheng Xie, Xinda Han, Zehao Wei, Pengpeng Zhang, Songyun Zhao, Xiumei Wang, Xufeng Huang, Min Xu

**Affiliations:** 1 Department of Gastroenterology, Affiliated Hospital of Jiangsu University, Jiangsu University, Zhenjiang, 212001, China.; 2 Digestive Disease Institute of Jiangsu University, Affiliated Hospital of Jiangsu University, Zhenjiang, 212001, China.; 3 Department of Oral and Maxillofacial-Head and Neck Oncology, Shanghai Ninth People's Hospital, Shanghai Jiao Tong University School of Medicine, College of Stomatology, Shanghai Jiao Tong University, Shanghai, China.; 4 Department of Plastic Surgery, Xiangya Hospital, Central South University, Changsha, China.; 5 Xinglin College, Nantong University, Nantong, Jiangsu, China.; 6 Department of Lung Cancer, Tianjin Medical University Cancer Institute and Hospital, Tianjin, China.; 7 Department of Neurosurgery, Wuxi People's Hospital Affiliated to Nanjing Medical University, Wuxi, China.; 8 Affiliated Cancer Hospital of Inner Mongolia Medical University, 010020, Inner Mongolia, China.; 9 University of Debrecen, Debrecen, Hungary.

**Keywords:** Innate lymphoid cells, Inflammation, tumor immune microenvironment, cytokine, immunotherapy, Digestive system

## Abstract

The incidence and mortality of digestive system-related cancers have always been high and attributed to the heterogeneity and complexity of the immune microenvironment of the digestive system. Furthermore, several studies have shown that chronic inflammation in the digestive system is responsible for cancer incidence; therefore, controlling inflammation is a potential strategy to stop the development of cancer. Innate Lymphoid Cells (ILC) represent a heterogeneous group of lymphocytes that exist in contrast to T cells. They function by interacting with cytokines and immune cells in an antigen-independent manner. In the digestive system cancer, from the inflammatory phase to the development, migration, and metastasis of tumors, ILC have been found to interact with the immune microenvironment and either control or promote these processes. The conventional treatments for digestive tumors have limited efficacy, therefore, ILC-associated immunotherapies are promising strategies. This study reviews the characterization of different ILC subpopulations, how they interact with and influence the immune microenvironment as well as chronic inflammation, and their promotional or inhibitory role in four common digestive system tumors, including pancreatic, colorectal, gastric, and hepatocellular cancers. In particular, the review emphasizes the role of ILC in associating chronic inflammation with cancer and the potential for enhanced immunotherapy with cytokine therapy and adoptive immune cell therapy.

## Introduction

### Tumor immune microenvironment and immunotherapy

Cancer is the second leading cause of death in the United States. In 2023, the estimated number of incidences and deaths by digestive system-related cancers was 348,840 and 172,010, respectively. Furthermore, the highest incidence and mortality rates were significantly attributed to colorectal cancer (CRC) and pancreatic cancer, respectively 1. Therefore, there is an urgent need for earlier diagnosis as well as more effective treatment modalities. The in-depth investigation of tumor mechanisms and the development of new technologies will offer additional strategies to fight cancer 2-4.

The complexity of the tumor immune microenvironment, along with immune escape and resistance of tumor cells, significantly limits the clinical efficacy of conventional therapies like surgery, chemotherapy, and radiotherapy 5. Targeting and precision are the new compasses of cancer treatment 6, 7. Immunotherapies, including modified cytokines, over-the-counter cell therapies, immune checkpoint blockade, and cancer vaccines, have emerged as new options for cancer control. The effectiveness of these immunotherapies is often correlated with prolonged survival and reduced recurrence rates 8, 9. Cytokines, secreted by inflammatory, tumor, or immune cells facilitate cellular interactions and communication as well as crucial mediators linking inflammation and cancer. Cytokine therapy aims to enhance growth-inhibitory and immunostimulatory cytokines such as interferon (IFN) and Interleukin-2 (IL-2), IL-7, IL-12, and IL-15. Furthermore, it also inhibits inflammatory and tumor-promoting cytokines like tumor necrosis factor (TNF), IL-1β, and IL-6 10. The cytokine therapies are continuously advancing, with IL-17, emerging as a key drug target for various chronic inflammatory diseases because of its modulatory effect on inflammation, resistance to pathogens 11, and therapeutic potential against numerous types of solid tumors 12, 13. Innovations in cytokine construction methods and delivery modalities are further revitalizing cytokine therapy. The use of antibody-cytokine fusion molecules in conjunction with chemotherapy has proven effective in treating CRC 14, 15. Moreover, the delivery capabilities of pluripotent stem cells and biomaterials for cytokines are under active development 16. Recently, an innate lymphocyte closely related to cytokines has been discovered to be associated with the dynamic progression from inflammation to tumor.

### Role of ILC in progression from inflammation to cancer stage in the digestive system

Innate lymphoid cells (ILC) are a family of immune cells found throughout the mucosal tissues of the gastrointestinal tract, pancreas, and liver. They help the body build a first line of defense against external stimuli. Unlike T cells, ILC lack the antigen-specific T cell receptor (TCR) and functions primarily by secreting cytokines and interacting with other immune cells. Based on the secretion of characteristic cytokines and dependent transcription factors, ILC are divided into three groups, including group I ILC [comprising Natural killer (NK) cells and type 1 ILCs (ILC1s)], and groups ILC2s and ILC3s. NK cells and ILC1s are dependent on T-bet and Eomes, respectively, and characteristically express IFN-γ and granzyme 17, 18, while ILC2s are dependent on GATA-3 and express IL-5, IL-9, IL-13 and amphiregulin (AREG) 19, 20. The ILC3s group depends on RORγt and secretes IL-17 and IL-22 respectively depending on the phenotype 21. ILC play a pivotal role in the regulation of the immune system and the maintenance of tissue homeostasis. ILC that secrete cytokines are involved in numerous immune pathways, including the control of immune responses to commensals and pathogens at the mucosal barrier, the enhancement of adaptive immunity, and the modulation of inflammatory responses. Comprehensive analysis of these cells revealed that they play different or even opposite roles in the dynamic procedure of inflammation to tumors in the digestive system 22-25. This contradiction might be because ILC are closely connected to their surroundings and have a high degree of plasticity 16. At different sites and stages, ILC are molded into distinct subpopulations that secrete different cytokines or exhibit specific interactions with surrounding cells.

Inflammation is primarily a defense mechanism of the body against external stimuli to maintain homeostasis. During acute inflammation, the inflammatory response initiates the repair by secreting inflammatory molecules and facilitating chemotaxis of inflammatory cells, such as macrophages and neutrophils, toward the site of injury for detoxification. However, when inflammatory molecules are overexpressed or signaling pathways related to anti-inflammatory responses and inflammation reduction are impaired, external stimuli are not eliminated within a short time, and ultimately the duration of inflammation extends, leading to the transition to chronic inflammation. This transition has been found to regulate cancer* via* different mechanisms such as chemotaxis, tumorigenesis, proliferation, vascular network formation, as well as invasion and metastasis. It has been observed that in the digestive system, controlling chronic inflammation is closely associated with prevention, suppression of progression, and better prognosis in colorectal, pancreatic, gastric, and liver cancers 26-29. Furthermore, chronic inflammation is an important target for cancer treatment and prevention 30. In addition to damaging DNA, promoting cell proliferation, and modulating signaling pathways, the tumor-promoting effects of chronic inflammation also depend on the construction of a suppressive tumor immune microenvironment. With the accumulation of immunosuppressive inflammatory factors 31, immunosuppressive cells are recruited 32, immune cells are then inhibited 33, and differentiated into immunosuppressive phenotype 34, ultimately the function of the inflammatory response is slowly shifted from defense and repair to cancer promotion. Currently, the studies on the digestive system-associated crosstalk between chronic inflammation and cancer have been focused on the effect of *Helicobacter pylori* (Hp) and related inflammation on gastric cancer, fibrosis in pancreatic and hepatocellular cancers, and the inflammatory response that damages the mucosal barrier of the gastrointestinal tract and promotes cellular chemotaxis 35-39. Considerable research suggests that ILC are associated with the key nodes described above by producing cytokines or engaging in communication with specific cells. Chronic inflammation and its related cytokines, ILC, and the tumor immune microenvironment form a network. Effective control of ILC are beneficial for limiting the inflammatory response toward inhibiting digestive system tumors. This paper discusses the role of ILC in digestive system inflammation to cancer progression, specifically CRC, gastric, liver, and pancreatic cancers. It was observed that the interaction between ILC, cytokines, and other immune cells is the key underlying mechanism.

## The role of ILC subsets

### NK cells and ILC1s

The closely related ILC1s and NK cells together constitute the first group of ILC. These cells co-express IFN-γ and granzyme, depend on transcription factors Eomes and T-bet for growth and development, and have similar surface markers and functional patterns. In contrast to ILC1s, NK cells do not depend on the transcription factor T-bet, instead, they require transcription factor Eomes for the maintenance of the identity and function 40. NK cells participate in the circulation and exert their potent cytotoxicity, playing an important role in innate immunity. Contrary to earlier beliefs that ILC1s are tissue-resident and non-cytotoxic, primarily modulate T helper type 1 (Th1) cells by secreting cytokines such as IFN-γ in response to Th1-like immune responses, recent studies have revealed that IL-15 mediates granzyme C expression and ILC1s cytotoxicity in a mouse breast cancer model, suggesting that ILC1s may also be cytotoxic 41. ILC1s and NK cells have been progressively characterized, and their tissue specificity has also been identified 42-44.

IFN-γ and TNF-α secreted by ILC1s are effective against multiple exogenous infections, such as cytomegalovirus, *Toxoplasma gondii*, and vaccinia virus 17, 45, 46. However, the excess production of IFN-γ by ILC1s can exacerbate colitis in Crohn's disease mice 47. Furthermore, intraepithelial lymphocytes contribute to the development of intestinal inflammation by supporting the survival of ILC1-like cells 48.

Whether NK cells specifically promote or inhibit cancer is closely correlated with their specific phenotype 49, 50. The data indicate that NK cell infiltration significantly modulates in solid tumors 50. Differing from CD56^dim^ NK cells, which are predominantly circulating and have a potent cytolytic capacity with killer immunoglobulin-like receptors, CD56^bright^ NK cells are primarily found in secondary lymphoid organs and tissues and play a pro-inflammatory role by secreting IFN-γ, granulocyte-macrophage colony-stimulating factor (GM-CSF), TNF, and IL-10 51. In some tumors, NK cells exhibit a specific phenotype resembling the tissue-resident NK (trNK) cell population. Among them, decidual NK (dNK) cells have been observed to release pro-angiogenic factors and altered effector functions that could potentially induce cancer growth 52. Another trNK-like tumor-infiltrating NK cell with antitumor functions has been identified as intraepithelial ILC1s (ieILC1s), which have been associated with malignancy suppression and can be stimulated by IL-12 and IL-15 to produce IFN-γ 53.

Cytokines and a high degree of plasticity further complicate the role of group I ILC in tumorigenesis, especially due to the ease of conversion of NK to ILC1s or some other immunocompetent phenotype. IL-15 and transforming growth factor-β (TGF-β) are strongly associated with the plasticity of group I ILC. For instance, in breast precancerous lesions as well as head and neck squamous cell carcinoma models, IL-15 mediates the production of granzymes by CD103^+^ cytotoxic ILC1s and promotes the proliferation of ieILC1s, which may inhibit cancer 54, 55. Another important cytokine, TGF-β, predominantly plays a pro-tumorigenic role* via* the NK cells to ILC1s transition 56. TGF-β can mediate the conversion of NK cells to intermediate type 1 innate lymphoid cell (intILC1s) populations and ILC1s populations, thereby enabling tumors to evade innate immune system surveillance 57. This transition is particularly evident in CD56^bright^ NK cells and can be augmented by IL-5, further highlighting the role of ILC's plasticity in their specific functions 58. Furthermore, ILC1s can differentiate into other ILC subtypes and cause different outcomes, as evidenced in squamous cell carcinoma and hepatocellular carcinoma (HCC) models 59, 60.

NK cells and ILC1 exert specific effects on the body in different microenvironments. ILC1s have a more complex type and function than NK cells in the traditional sense of killing cells and are associated with but not limited to factors like IL-15 and TGF-β. A more systematic phenotyping of these cells and the effect of cytokines on their plasticity will potentially offer insights into new treatments for cancer or inflammation.

### ILC2s

Transcription factor GATA-3 is a key factor that aids ILC2s in differentiation, maintenance, and function 61. Similar to group 1 ILC, the phenotype and function of ILC2s are tissue-specific 62. ILC2s are not only tissue-resident but can also participate in innate type 2 immune and inflammatory responses in the circulation 63. As the counterpart of Th2 cells, ILC2s produce type 2 cytokines (IL-4, IL-5, IL-9, and IL-13) and are implicated in parasite immunity, airway inflammatory diseases, tissue repair, and tumor regulation. Furthermore, ILC2s are crucially associated with different mechanisms of immune defense against various parasites such as by facilitating pathogen efflux and migrating inflammation to lymphoid tissues 64, 65. Moreover, ILC2s are closely associated with allergic airway diseases such as asthma, and their independent secretion of IL-5 and IL-13 confirms their important role in airway inflammation 66. Transcription factor RORγ induced ILC2 secretion of IL-17A, IL-17F, and IL-22 in chronic airway inflammation 67. IL-2 signaling enhances ILC2 survival and proliferation and aids in the production of type 2 cytokines to promote eosinophilic pneumonia 68. It has been observed that ILC2's functions are modulated by their specific environment. In addition, ILC2s are not only modulated by cytokines but certain lipid molecules have also been observed to play a significant role. IL-4 and IL-33 work synergistically in mediating ILC2 proliferation and promoting type 2 cytokine production 69. Whereas, IFN-γ and IL-27 negatively regulate tissue-resident ILC2s 70. Studies have shown that prostaglandin E2 can inhibit ILC2 activation 71. ILC2s are also instrumental in tissue repair. Increased levels of IL-33 in wounds increase ILC2 cells, thereby enhancing wound healing in mice 72.

The tumor microenvironment plays a crucial role in determining the differential impact of ILC2s on tumors. Moreover, IL-33 is a key mediator of most ILC2s-associated tumor immunity and ILC2s can even play an opposite role 73-76. Other than cytokines, ILC2s rely on interactions with other immune cells in the tumor microenvironment. ILC2s have also been observed to interact with other immune cells, such as in airway inflammation, they can interact with T cells, DC, macrophages, mast cells, or basophils, whereas in cancer, they may interact with myeloid-derived suppressor cells (MDSC), regulatory T cells (Tregs), eosinophils, and neutrophils. Tregs and MDSC are recognized as key immunosuppressive cells that play a negative role in cancer defense. The suppressive function of Tregs is enhanced by ILC2s production of AREG protein, promoting EGFR-expressing tumor progression 77. IL-13-producing ILC2s may further promote immunosuppression from MDSCs during advanced stages of breast cancer metastasis 78. IL-33-induced amplification of ILC2s inhibits the activation of NK cells and their cytotoxic effects, thereby promoting tumor growth 79. Overall, although various significant mechanisms of ILC2s have been extensively studied, many details still require further exploration to fully understand their bidirectional role in various diseases.

### ILC3s

ILC3s, which mirror Th17/Th22 cells, consist of several components: NCR^+^ ILC3s, NCR^-^ ILC3s, and lymphoid tissue inducer (LTi)/LTi-like cells. In mice, NCR^+^ ILC3s are NKp46^+^, whereas in humans, NCR^+^ ILC3s are NKp44^+^. ILC3s in mice produce both IL-17 and IL-22, whereas in humans NKp44^-^ ILC3s secretes IL-17 and NKp44^+^ ILC3s produce IL-22. The development of NCR^+^ILC3s and LTi cells as well as cytokine production, significantly rely on RORγt 80. Other than survival, RORγt and RORα are intrinsically linked with the functional maintenance and proliferation of ILC3s 81. The Aryl hydrocarbon receptor (AHR) is another crucial transcription factor for ILC3s, which interacts with the Notch signaling pathway. A deficiency in AHR leads to fewer RORγt^+^ ILC with diminished IL-22 production, thus inhibiting protection against intestinal bacterial infections 82. ThPOK is a novel key factor that regulates NKp46^+^ ILC3s differentiation and is significantly associated with the maintenance of ILC3s function and lineage stability 83. LTi cells are integral in secondary lymphoid organ formation through Lymphotoxin β-receptor signaling, a feature absent in LTi-like cells 84. Moreover, ILC3s can also undergo conversion to other ILC subpopulations. Recent research indicates that T-bet promotes the transformation of human ILC3s into functional NK cells 85.

ILC3s are associated with gastrointestinal homeostasis, inflammatory diseases, and cancer, and their function is dependent on bacteria. In the gastrointestinal tract, ILC3s are primarily involved in barrier repair and the inhibition of bacterial translocation, contributing to the maintenance of homeostasis. In addition, ILC3s strengthen the gastrointestinal barrier by secreting IL-13 86. They also eliminate circulating microorganisms by inducing the production of antimicrobial peptides in the liver, which aids in postoperative liver regeneration 87. The high-fat diet-induced hepatocyte apoptosis can be mitigated by the upregulation of hepatic lipid metabolism following the macrophage-induced proliferation of ILC3s *via* IL-1 88. IL-22, secreted by ILC3s, is crucial in maintaining intestinal barrier function and facilitating interactions with bacteria. IL-22-mediated mucus multiplication provides an early defense against pathogens 89. Short-chain fatty acids (SCFA) play a crucial role in intestinal mucosal homeostasis and ILC3s in the colon have numerous SCFA receptors on their surface. The congenital absence of these receptors impacts ILC3s' proliferation and IL-22 production, which reduces immunity against colony infections and aids tissue repair 90, 91. Whereas when the secretion of IL-22 by CD4^+^ T cells and ILC3s is enhanced by SCFA, intestinal infections can be better controlled 92. The relationship between ILC3s and intestinal flora is bidirectional and contact with the intestinal flora is essential for the development and function of ILC3s. It has been observed that SCFAs are a major metabolite of microorganisms which has indicated that microorganisms can activate ILC3s through direct contact. Tryptophan, a digestive product of the intestinal microbiota and a ligand for the AHR, supplies energy to Lactobacillus spp. Furthermore, AHR is a key transcription factor that supports the function and differentiation of ILC3s. Microbiota can also activate ILC3s directly, such as by mediating IL-1β and IL-23.

The literature has indicated that inflammation crucially modulates the association between inflammatory bowel disease (IBD) and ILC3s. During inflammation, ILC3s play a protective role and support the intestinal barrier; however, chronic inflammation can convert ILC3s into a pro-inflammatory phenotype. In this state, they express additional inflammatory factors, creating a positive feedback loop that exacerbates intestinal inflammation 93. Apart from its protective role against intestinal bacterial infections, IL-22 may also have detrimental effects on IBD. In an anti-CD40 antibodies-induced acute congenital colitis model, IL-22 secreted by ILC3s was found to aggravate inflammation 94.

ILC3s have different impacts on tumors, including direct contact, cytokine production, and the formation of tumor-associated tertiary lymphoid structures (TLS), attributed to their significant immune recruitment capacity. Furthermore, ILC3s have been shown to exert antitumor effects on HCC and melanoma cells* via* IFN-γ release and Tumor necrosis factor-related apoptosis-inducing ligand (TRAIL)-dependent cytotoxicity stemming from direct recognition 95. Whereas IL-22 from ILC3s has been linked with cancer promotion. In a colitis-associated carcinoma mouse model, IL-22 induced epithelial signal transducer and activator of transcription 3 (STAT-3) phosphorylation and proliferation, thereby facilitating cancer development 96. The NLRP3 and NLRP6 inflammasomes are known to reduce IL-22 binding protein levels, leading to an uncontrolled increase in IL-22 and subsequent stimulation of colon cancer 97. Moreover, ILC3s have also been observed to promote cancer *via* the IL-23/IL-17 axis. IL-23 activates ILC3s, contributing to intestinal carcinogenesis even in the absence of carcinogens 12. IL-17 enables tumors to develop resistance to vascular endothelial growth factor inhibitors, further promoting angiogenesis and tumor growth 98. Similar to secondary lymphoid organs, LTi cells have been observed to mediate TLS formation and positively influence survival and prognosis 99. LTi cell formation is associated with immune recruitment, which broadly influences the function of ILC3s. Upon activation by IL-12, ILC3s, expressing various chemokine receptors, can potentially inhibit tumor growth by recruiting numerous immune cells such as CD8^+^ T cells, activated MDSC, NK cells, and natural killer T (NKT) cells. However, this is countered by plasmacytoid pre-DC inducing ILC3 apoptosis *via* the CD95-mediated pathways 100. This recruitment may also unintentionally support cancer development. In addition, the recruitment of NKp44^-^ ILC3s, Tregs, and MDSC by Chemokine ligand 21 (CCL21) promotes the establishment of a tumor-supportive environment 101. The specificity of ILC3s depends on the type of cancer, the stage of tumor development, and the characteristics of the immune microenvironment. Therefore, because of ILC3s' intricate crosstalk with various microbiomes and the environments they inhabit, they are a potential therapeutic target.

## ILC act as a bridge between inflammation and tumor

### Prolonged inflammation can also mark the beginning of cancer

Inflammation is a protective process of the body against external stimuli such as infection or injury, which repairs the damage and limits infection. Inflammatory responses are categorized into acute and chronic inflammation depending on their duration. Acute inflammation is characterized by the migration of macrophages and neutrophils to the site of injury and the secretion of chemokines and cytokines, which ultimately leads to the removal of exogenous substances and healing of the injury. This phase is characterized by a rapid and intense inflammatory response, and after the inflammatory cells have completed their mission, they are either eliminated or undergo apoptosis 102. To facilitate this resolution, specialized pro-resolving mediators inhibit neutrophil tissue infiltration, mediate apoptosis, remove stimuli, and modulate cytokine secretion 103.

However, not all inflammation can be controlled and restored to homeostasis. Chronic inflammation is an excessive and deleterious response that initiates for self-defense and repair and is characterized by the accumulation of M2 macrophages, MDSC, Treg cells, and inflammation-suppressing cytokines, which establishes an immunosuppressive microenvironment. Although the involved mechanisms are still being investigated, the link between chronic inflammation and tumor development is indisputable. Chronic inflammation supports tumor development by damaging DNA, promoting cell proliferation, and modulating signaling pathways, thereby contributing to tumor progression, distant metastasis, and resistance to therapy. Furthermore, the DNA damage caused by inflammation further stimulates inflammation, creating a positive feedback loop that increases the frequency of mutations. In addition, inflammation-induced cell proliferation and migration support tumor development 104. Inhibition of inflammatory response* via* peroxisome proliferator-activated receptors is a promising therapeutic strategy that inhibits tumor cell growth and proliferation 105. The nuclear factor κB (NF-κB)-related signaling pathway is a bridge between inflammation and cancer and is associated with many cancer-related genes. Moreover, it has various roles, such as induction of pro-proliferative and anti-apoptotic genes, modulation of other signaling pathways, influencing genetic, epigenetic, and cellular metabolic changes, promotion of epithelial-to-mesenchymal transition, tumor invasion, and angiogenesis support, cancer metastasis, and even treatment tolerance and immunosuppression 106, 107. The zinc finger transcription factor Miz1 is a newly identified tumor suppressor that inhibits liver tumorigenesis, possibly by isolating oncoprotein metadherin, thus preventing metadherin from activating NF-κB, and by shifting the macrophage phenotype toward a pro-inflammatory phenotype 108.

### ILC shape immune microenvironment to modulate the progression of inflammation to cancer

The link between inflammation and cancer extends beyond the above modalities, and their progression is significantly linked with induced immunosuppression. Chronic inflammation can create an immunosuppressive microenvironment either by increasing immunosuppressive inflammatory factors, enhancing the concentration of immunosuppressive cells, reducing the efficacy of immune cells, or by promoting the differentiation of otherwise neutral immune cells towards an immunosuppressive phenotype. This chronic inflammation-induced immunosuppressive microenvironment is either suppressed or enhanced by ILC, potentially because of its tight association with cytokines and immune cells. Cytokines specifically secreted by various ILC subpopulations have been indicated to be involved in the progression from chronic inflammation to cancer. It has been observed that NK cells and their secretion of IFN-γ inhibit the progression from chronic pancreatitis to pancreatic cancer by inhibiting hepatic stellate cells (HSC) and the fibrosis they cause 39, 109, 110. The IgA response due to ILC2s-induced IL-5 is important for the clearance of HP and the prevention of gastritis transformation to gastric cancer 35, 111. ILC3s and their secretion of IL-22 have been shown to induce the progression of chronic inflammation to colon cancer and association with the perpetuation of colon cancer 96. Moreover, other than cytokines, ILC significantly regulates the tumor immune microenvironment by interacting with immune cells. These crosstalks are multifaceted, specifically in immune cell recruitment, influencing immune cell activity and function, and shifting the direction of immune cell polarization.

Chemokines are widely upregulated as a specific type of cytokine in the inflammatory response. Furthermore, chemokines mediate the migration of immune cells involved in the development of anti-tumor immunity or induce a pro-tumor microenvironment, Moreover, they are also recognized as biomarkers with diagnostic effects that can predict efficacy and prognosis 112. For instance, CCL15 can recruit monocytes, eosinophils, and neutrophils, is a prognostic marker for HCC, and is associated with metastasis 113-115. In head and neck squamous cell carcinoma, CCL15 induces gefitinib resistance *via* NF-κB 116. Increased expression of CCL-16 by centrosomal P4.1-associated protein underpins inflammatory pathways and HCC 117. Chemokines recruit ILC and their recruitment of immune cells is also important for shaping the tumor immune microenvironment. CCL2 induces the recruitment of ILC3s to tumors and stimulates the production of CXCL13 by tumor stromal cells, which ultimately leads to the production of the cancer cell motility factor receptor activator of NF-κB ligand and promotes lymphatic metastasis of breast cancer cells 118. However, in the breast cancer tumor microenvironment, CX3CL1 overexpression attracts NK cells, which then inhibits tumor growth and metastasis, and thus improves the efficacy of trastuzumab in the treatment of low-expressing human epidermal growth factor receptor 2 cancers 119. IL-21-stimulated NK cells can secrete more chemokines and recruit activated T cells, thus enhancing the therapeutic effect of pancreatic cancer 120. Similarly, the recruitment of DC by ILC2s has been observed to activate T-cell immunity and suppress pancreatic cancer 77. CXCL2 derived from ILC2s recruits neutrophils and promotes HCC progression 121. GM-CSF and IL-5 produced by ILC2 exert anti-melanoma effects by recruiting eosinophils 122.

The effects of ILC on the activity and function of immune cells are also crucial for the tumor immune microenvironment. T cells and DCs, which are antigen-presenting, play an important role in fighting cancer and modulating inflammation. However, chronic infections and cancer lead to the production of depleted T cells with reduced proliferative capacity and effector function 123, 124. Several studies have suggested that highly correlated ILC may be important in regulating T-cell immunity. NK cells have multiple pathways for DC recruitment, promotion of DC maturation, killing of immature DC, and inhibition of DC crossing over to enhance the antitumor effect of T cells 125-128. IL-9 secreted by ILC2s correlates with T-cell activation and CRC suppression 129. Furthermore, IL-13 secreted by ILC2s activates MDSCs and subsequently negatively regulates T-cell proliferation 130. The T cells exerting anti-tumor effects as described above are CD8^+^ T cells, while Treg cells are a specific type of immunosuppressive T cells, that also interact with ILC. The enhanced Treg cell function induced by ILC2s secreted AREG can markedly promote CRC progression 131. In contrast, secretion of GM-CSF by ILC3s can reduce the number of Tregs in the gut 132. It is well established that MDSC acts as one of the major players in suppressing tumor immunity 133. In addition to attracting MDSC to the tumor immune microenvironment, ILC can also enhance MDSC function. ILC3s can be shaped into secreted ILCreg to further enhance the recruitment effect while secreting IL-17 and IL-22 for MDSC recruitment 134. IL-13-producing ILC2s may further promote MDSC-mediated immunosuppression during advanced stages of breast cancer metastasis 78.

It has been observed that in addition to Treg and mast cells (immunosuppressive) or NK and CD8^+^ cytotoxic T lymphocytes (immune-promoting), the role of some immune cells, such as macrophages, neutrophils, and DC might be polarized, and depends on chronic inflammation 135. Specifically, the conversion of macrophages to the M1 phenotype is associated with inhibition of tumor progression, while the shift to the M2 phenotype suggests tumor growth and invasion. This data reveals the possibility of using macrophages to regulate cancer development and regression 136, 137. Different ILC subpopulations are involved in their polarization and may influence the direction of polarization. Studies have shown that NK cells are beneficial in controlling fibrosis, an important step in hepatitis leading to HCC, possibly by polarizing macrophages to an M1 phenotype during the hepatitis phase 138. Furthermore, adipose-resident ILC1 populations can drive the shift of macrophages toward a pro-inflammatory M1 phenotype 139. Increased M2-type macrophages caused by ILC2s mediate an immunosuppressive microenvironment in gastric cancer 140. GM-CSF derived from ILC3s promotes macrophage polarization toward the M1 phenotype and stimulates self-activation through a positive feedback loop, helping to maintain intestinal homeostasis and inhibit fibrosis 141. In turn, macrophage polarization influences ILC function. IL-1β secreted by M1-type macrophages determines the production of ILC2-derived IL-22, which contributes to the repair of inflammatory injury 142.

Overall, the bridge between inflammation and cancer is built by the constant interaction of ILC with cytokines and immune cells. Moreover, ILC themselves are shaped in their process of shaping the tumor immune microenvironment.

The following section will elaborate on how ILC regulate the progression of inflammation to cancer in the digestive system.

## Clues from digestive system inflammation to tumor-ILC

### Pancreatic disease

Pancreatic adenocarcinoma, mainly pancreatic ductal adenocarcinoma (PDAC), is characterized by malignant features such as early metastasis, which poses a challenge for early diagnosis, and limits therapeutic efficacy. Despite the advent of chemotherapy, surgery, immunotherapy, targeted therapy, and combination therapy, its high incidence and the fact that it is still the fourth most common cause of cancer mortality underscore the importance of continued and in-depth study 1. Chronic pancreatitis is a progressive, irreversible, multifactorial fibroinflammatory disease. Furthermore, it has been observed that chronic pancreatitis-mediated inflammatory response and metaplasia of alveolar cells to the ducts are crucial mediators of the transition from pancreatitis to pancreatic cancer. The previous literature was primarily focused on oxidative stress, cytokines, shared inflammatory signaling pathways, and the transformation of pancreatic stellate cells and alveolar cells 143-145. However, the importance of ILC was observed after multiple ILC phenotypes were identified in pancreatic diseases.

The largest proportion of NK cells are active in both pancreatic cancer and pancreatitis. Activated CD56^+^ NK cells are clustered in the pancreas of patients with chronic pancreatitis, and NK cells directly correlate with the benign outcomes of survival and recurrence in patients after pancreatic cancer surgery 146, 147. NK cells can induce cancer stem cell (CSC) differentiation and inhibit pancreatic tumor growth and metastasis by secreting IFN-γ and TNF-α 148. IL-21 enhances the antibody-dependent cell-mediated cytotoxicity (ADCC) of NK cells and stimulates their secretion of IFN-γ and chemokines for recruiting activated T cells, and its combination with cetuximab reduces tumor load in mice 120. IL-2 is an important anti-tumor growth factor. The targeted immune cytokine L19-Interleukin-2 (L19-IL2) induces extensive necrosis and proliferation restriction in pancreatic cancer with the indispensable role of NK cells 149. Furthermore, IL-15 has been observed to induce NK cell growth and proliferation and exert antitumor activity 150. Moreover, NK cells and recombinant IL-15 (rIL-15) can inhibit fibrosis in chronic pancreatitis, a key target for pancreatitis and pancreatic cancer treatment, by inhibiting the atrophy of alveolar cells, preventing tissue collagen accumulation around blood vessels, and regulating pro-fibrotic genes 36, 151. IL-15 in combination with CD40 agonists showed significant pancreatic cancer suppression, which was also associated with NK cells 152. ILC2s are another major mediator in pancreatic cancer with a dual nature predominantly dependent on its activator, IL-33. On one hand, IL-33 induces ILC2s-mediated recruitment of DC and subsequent recruitment and activation of CD8^+^ T cells, and on the other hand, ILC2s also have the ability to enhance the blockade of the programmed cell death protein-1 (PD-1) pathway. Therefore, immunotherapies co-targeting ILC2s and T cells warrants further research 74. However, intra-tumoral fungi mediate IL-33 secretion by PDAC cells, and genetic defects in IL-33 or antifungal treatment inhibit pancreatic tumor growth and support prolonged mouse survival 73. This paradox in turn may be related to the high plasticity of ILC2s. Recently, it was observed that under hypoxic conditions, ILC2s transform into immunosuppressive ILCreg. In a subcutaneous PDAC tumor mice model, the injection of hypoxia-treated ILC2 cells significantly increased tumor volume and subsequent peritoneal metastasis. Through RNA-seq sorting of ILC2s from hypoxic mice at various time points, researchers concluded that hypoxia induces reversible differentiation of ILC2s into high IL-10 expressing ILCreg, and this immunosuppressive effect can be counteracted by neoadjuvant AG (nab-paclitaxel + gemcitabine) 34. In addition, IL-22 secreted by ILC3s mediates AKT signaling and promotes pancreatic cancer progression and metastasis, whereas concomitant IL-22/IL-22R or AKT blockade has also been shown to be effective 153. However, the presence of intra-tumoral TLS promotes better prognosis for many tumors, including pancreatic cancer, and targeting TLS formation might also be a novel idea for immunotherapy 154. Previous data have confirmed that the levels of NCR^+^ ILC3s in non-small cell lung cancer are positively correlated with disease stage and are associated with the density of TLS in the tumor 155. Therefore, targeting ILC3s may inhibit the induction of TLS formation in pancreatic cancer.

Altogether, under the mediation of various triggers, ILC play a crucial role in the prolongation of chronic pancreatitis as well as the development and regression of pancreatic cancer. This is achieved through the secretion of cytokines, regulation of signaling pathways, enhancement of self-efficacy, or chemotaxis of other immune cells. These multifaceted interactions highlight the complexity and significance of ILC in the pathophysiology of pancreatic diseases.

### Intestinal diseases

In the United States, both new cases and deaths related to CRC have been ranked third among all cancers 1. Colon cancer is a disease that is highly influenced by external environmental and genetic factors. The molecular mechanisms of CRC have been partially elucidated, including the most common chromosomal instability, microsatellite instability due to DNA mismatch repair, and the CpG island methylation pathway. However, although understanding the mutation mechanism is crucial, there are various other factors involved. The association between CRC and inflammation has been increasingly recognized 156, 157. Recent studies have indicated an increased risk of CRC in people with IBD as the inflammatory response has been observed throughout the development of CRC. Firstly, chronic inflammation initiates and promotes tumorigenesis by inducing DNA damage or epigenetic changes. Secondly, inflammation is secondary to tumors, and factors like hypoxia-induced cell death or disruption of the epithelial barrier, as well as invasion of microbial products, contribute to cancer development. Finally, pro-tumorigenic inflammation is often caused by treatment-induced necrotic cells that produce damage-associated molecular patterns 158. Furthermore, the role of ILC in intestinal inflammation and their crosstalk with intestinal microorganisms have been extensively studied, suggesting that a deeper understanding of ILC could lead to novel approaches in CRC treatment.

The NK cells and ieILC1s make up the largest proportion of innate lymphocytes that infiltrate CRC cells 156. NK cells have a suppressive effect on intestinal inflammation and CRC; however, the definitive mechanism for this effect remains unclear and might be achieved* via* other immune cells 159-161. However, in some cases, the inhibitory function of NK cells against CRC is limited or even reversed, which may be attributed to the specialized phenotype of NK and ILC1 cells and their function being influenced by the tumor microenvironment 162. The studies have observed that after co-culture with tumor cells, the degranulation capacity and ability to release cytotoxic molecules and IFN-γ were decreased in NK cells 163. NK Cells transform to a dNK phenotype and promote CRC progression by secreting pro-angiogenic cytokines 164. Moreover, in the intestinal inflammatory environment, ILC1s cause extracellular matrix remodeling, which contributes to fibrosis and tumor growth 165. In addition, the loss of ILC1s due to T-bet depletion provides a defense against severe colitis in mice 24. Overall, the current research is not sufficient to draw specific generalizations about the role of group I ILC in CRC.

ILC2s have a dual role in both intestinal inflammation and intestinal tumors. These cells are crucial in protecting the intestinal tract. It has been observed that in the fight against amebic colitis, IL-33-mediated protection requires the presence of ILC2s 20. Mice lacking ILC2s acquired a higher tumor load, and high expression of ILC2s signature genes in tumors has been associated with a better prognosis in CRC 166. ILC2s are more abundant in CRC tissues than in adjacent tissues, and their IL-9 derivatives activate CD8^+^T cells and inhibit CRC growth 129. Furthermore, AREG may play a role in the interaction between ILC2s and intestinal inflammation. Neurohormone U (NMU) mediates the production of AREG in ILC2s and is associated with the intestinal barrier, inflammatory protection, and anti-parasite activities 167. However, previous studies have shown that high levels of AREG positively correlate with CRC malignancy. AREG derived from ILC2s suppresses tumor immunity by enhancing the function of Tregs 131. AREG function has been observed to change as inflammation progresses to cancer, which requires comprehensive research. STAT6 is a molecule that is positively associated with intestinal tumor formation in mice and the progression and poor prognosis of humans. Its level is downregulated in the epithelium after it neutralizes ILC2s-derived IL-13 to modulate intestinal permeability and DS-induced colitis 168. IL-25 promotes ILC2s in tumors, maintaining tumor-infiltrating MDSC to form an immunosuppressive tumor microenvironment 169. Moreover, as an activator of ILC2s, IL-33 has been associated with CRC. The blockade of the IL-33/ST2 signaling axis relieves the inhibition of colon cancer 170. Conversely, IL-33 contributes to intestinal polyposis and tumorigenesis through immune and wound-healing responses mediated by tumor epithelial cells 76. The IL-33 receptor ST2 is highly expressed in colonic Treg cells and enhances their function 171. Overall, analyzing the targeting axis from ILC2 activators to products and their branches is necessary to further assess the role of ILC2s in CRC.

As the most prevalent ILC in the intestinal tract, ILC3s play a crucial role in maintaining the intestinal barrier, resisting pathogen infection, and cooperating with other immune cells. It has been noted that IL-22 secreted by ILC3s is crucial for the intestinal barrier and resistance to pathogens; however, they may have negative effects on intestinal inflammation such as IBD. This could be related to epithelial inflammation caused by endoplasmic reticulum stress response, necessitating the fine-tuned regulation of IL-22 to ensure beneficial outcomes 172. In colon cancer, IL-22 is mainly produced by NCR^+^ILC3s and then induces STAT-3 phosphorylation and proliferation by interacting with IL-22R on epithelial cells 96. During intestinal inflammation, IL23-reactive ILC3s increase and secrete IL-17, IL-22, and IFN-γ to mediate innate colitis 173. Furthermore, the signal transduction of microbial products drives IL-23 to stimulate tumor-induced inflammation and IL-17 response, thereby promoting tumor development, which may also be related to ILC3s 12. The plasticity of ILC3s supports its diverse roles. When ILC3s infiltrate into the tertiary lymphoid structure of the tumor, they differentiate into an ILC1s phenotype, and the subsequent loss of major histocompatibility complex class II and reduced communication with T cells may be key to their reduced immunity 174. TGF-β signal transduction initiates the transformation from ILC3s to ILCreg 175. Therefore, constructing a more detailed ILC-cytokine-tumor immune microenvironment network is essential to identify suitable therapeutic targets.

### Gastritis and gastric cancer

Gastric cancer, predominantly adenocarcinoma, is a prevalent malignancy in the digestive system. It ranks as the sixth most commonly diagnosed cancer and the fourth leading cause of cancer-related deaths 176. The risk factors for gastric cancer include HP, family history, poor diet, alcoholism, smoking, and Epstein-Barr virus infection 177. The HP-induced chronic non-atrophic gastritis leads to intestinal epithelial hyperplasia, which increases the risk of gastric carcinogenesis 35. Eradication of HP infection, particularly in first-degree relatives with a family history of gastric cancer, significantly reduces its incidence 178.

Multiple ILC subgroups, especially ILC2s, engage in crosstalk with HP, which diversely influences the progression of gastric cancer. Reduced abundance of NK cells has been observed in the tumor microenvironment of gastric cancer, which correlates with a worse prognosis 179. Additionally, NK cell function is often compromised or even reversed in gastric cancer. Persistent IFN-γ responses from NK and T cells post-HP infection may paradoxically enhance the risk of both gastric cancer and ulcers 180. HP-derived lipopolysaccharide promotes the proliferation of low cytotoxic IL-10-secreting NK cells, thus impeding the elimination of HP and fostering chronic infection 181. Other HP components, such as HopQ outer membrane adhesin and pro-inflammatory peptides, further impair NK cell functionality, adversely affecting gastric cancer prevention 182, 183. Moreover, the impact of ILC2s on HP can vary with the disease's duration. ILC2s are a predominant ILC subset in the stomach, which are quickly induced by HP infection, peaking at two weeks. Furthermore, ILC activation by IL-33, originating from commensal microbes, produces IL-5, which stimulates IgA release. This ILC2-dependent IgA response is critical for eliminating pathogenic HP, thus safeguarding the stomach 111. However, chronic HP infection suppresses IL-33 production, crucial for the effective activation of both ILC2s and T cells in the gastric mucosa and for inducing a Th2 response. Whereas exogenous IL-33 supplementation induces M2 macrophage polarization and transmural inflammation 22. Lastly, Connexin43, essential for gastric mucosal gap junctions, shows a positive correlation with the progression from inflammation to gastric cancer. Its significance in HP-associated gastric cancer is linked to an upsurge in the transcription factor GATA-3, an ILC2s marker 184.

ILC is not only associated with HP, but also affects gastric cancer by interacting with inflammatory molecules, hormones, chemotaxis, and cell polarization. This multi-faceted interaction highlights the complexity of gastric cancer pathology. Gastric cancer cell-derived prostaglandin E2 inhibits NK cell proliferation and promotes apoptosis 185. IFN-γ induces high 927 gene expression in gastric cancer cells with enhanced invasion and metastasis and reduced susceptibility to NK cells 186. Several cytokine therapies aiming to target and mitigate the adverse effects of NK cells have been explored. IL-2 helps NK cells restore Herceptin-mediated ADCC damage in gastric cancer patients 187. Recombinant mouse IL-15 (rmIL-15) facilitates NK cell proliferation and produces more IFN-γ to improve survival and inhibit liver metastasis in an inhibited gastric cancer mouse model 188. NKG2D extracellular domain assembles with human IL-15 to form a fusion protein, dsNKG2D-IL-15, which helps NK cells infiltrate gastric cancer tissues and inhibit the growth of gastric cancer in mice 189. Tumor cell-expressed CX3CL1 recruits NK cells and is associated with a better prognosis in gastric adenocarcinoma 190. Cytokine therapy is significant for reversing NK cell dysfunction in gastric cancer, which may be a novel therapy for this cancer. It has been identified that ILC2s are positively associated with the development of gastric cancer. Spasmolytic polypeptide-expressing metaplasia (SPEM) is associated with the pathological progression of intestinal-type gastric cancer. This association highlights the potential therapeutic targets in SPEM-related pathways. ILC2 depletion inhibits post-injury chemotaxis by inhibiting the reprogramming of principal cells into SPEM, which begins after acute mural cell loss 25. Endogenous intragastric glucocorticoids are necessary to inhibit the transcription of pro-inflammatory genes and the development of SPEM in the stomach 191. Recent studies suggest that this inhibition may be synergistic with the action of androgens on ILC2s. The study confirms that androgens prevent gastric inflammation and chemotaxis by blocking the production of pro-inflammatory cytokines of ILC2s, which is supported by the prevention of SPEM development through the depletion of ILC2s 192. Increased ILC2s in the periphery of gastric cancer patients and their resulting dysregulation of Th1/Th2 responses as well as increased MDSC and M2 phenotype macrophages constitute an immunosuppressive microenvironment in gastric cancer 140. Compared to the healthy individuals, the levels of ILC3s and IL22 were observed to be significantly increased in gastritis, and precancerous lesion patients, suggesting that ILC3s may also be associated with gastritis and gastric cancer 193. Polymorphisms in the IL-17 gene may also be associated with gastric cancer risk 194. However, experimental studies on ILC3s and gastric cancer are still lacking.

Overall, ILC and gastric cancer crosstalk involve HP infection, chemotaxis, microenvironmental shaping of ILC, and cytokine networks. Future research focusing on these interactions could pave the way for novel therapeutic strategies. More adequate studies could help to stifle gastric cancer at the primary stage or inhibit stage progression.

### Chronic hepatitis and liver cancer

Although liver cancer does not have a significantly increased incidence, it has the lowest five-year survival rate of all cancers and it ranks fifth in cancer-related deaths among men 1. HCC is more closely related to chronic inflammation than other cancers, and most HCCs are associated with chronic liver inflammation and its mediated cirrhosis 195-197. This strong link to inflammation highlights the critical need for targeted therapeutic strategies. Hepatic fibrosis as a pathological state is common in various viral hepatitis, non-alcoholic steatohepatitis (NASH), and alcoholic liver disease. The effectiveness of vaccines and antiviral treatment for some hepatitis is significant, but not sufficient. This gap necessitates the development of additional treatment options. Since liver fibrosis is generally considered irreversible, the main goal is to inhibit its progression. Immunotherapy for whole-body treatment has been recognized and promoted 198. As an emerging immune cell, the role of ILC in HCC has also been widely investigated. This section will focus on the role of ILC in liver inflammation, fibrosis, and interaction with the tumor microenvironment.

NK cells, which constitute approximately half of the lymphocytes in the liver 199, are thought to have the ability to inhibit hepatic fibrosis. HSC activation promotes the proliferation of fibrotic myofibroblasts is the initiation as well as the key process of fibrosis. In the early stage of liver fibrosis, activated HSC is recognized by NK cells, which subsequently mediate apoptosis through the TRAIL/FasL pathway, granule cytotoxicity, and the production of IFN-γ leading to HSC death 39, 109, 110. This demonstrates the potential therapeutic value of NK cells in early-stage fibrosis. Moreover, as fibrosis progressed to a later stage, HSC inversely inhibited the antifibrotic capacity of NK cells in a TGF-β-dependent form 23. NK-derived IFN-γ facilitates macrophage polarization to the M1 phenotype and prevents the progression of NASH to fibrosis 138. A recent study showed that CD8^+^ tissue-resident memory T cells induced hepatic stellate cell apoptosis to regress liver fibrosis in NASH 200. In addition, group 1 ILC inhibit the proliferation of CD8^+^ T cells by affecting the availability of IL-2 in long-term interaction with CD8^+^ T cells, which may be another target for inhibiting liver fibrosis 201. In the early HCC stage, inflammatory cytokines (such as TNF-α and IFN-γ) induce CD56^+^ NK cells to infiltrate tumors and kill cancer cells by stimulating chemokines production (such as CXCL10, CCL5, and CCL2) 202. The role of NK cells in HCC highlights their importance in both anti-fibrotic and anti-cancer responses. Specific localization of NK cells in HCC progression correlates with the respective phenotype of each subtype. The immune microenvironment of HCC is inextricably linked to the phenotypic shaping of liver-specific ILC. In NK cells IL-10 may excessively express NKG2A, which is positively correlated with NK cell depletion and dysfunction 203. *In vitro* studies have indicated that high TGF-β concentrations mediate the differentiation of NK-like cells to ILC1s as well as the loss of its cytotoxicity, whereas cytokine gradient mediates tumor ILC composition 204. Some immune cells can also affect NK cells. The activation receptor for NK, NKp30, reduces significantly after co-culture with MDSC, which inhibits both NK cytotoxicity and IFN-γ release 205. In addition, patients on immune checkpoint inhibitors indicated increased KLRF1^high^ NK-like ILC subgroup, which was associated with progression- and relapse-free survival, and warrants further research to assess the exact mechanism 206. CD49a^+^Eomes^+^ NK cells, typical dNK cells, can promote HCC development by promoting angiogenesis and reducing cytotoxicity, as also observed in CRC 207.

ILC2s promote chronic hepatitis, which may be associated with two cytokines, IL-33 and IL-13. Hepatocyte damage releases large amounts of IL-33 to promote ILC2s activation and expansion, while IL-13 secreted by ILC2s promotes hepatic fibrosis in a STAT6-dependent manner or by activating HSC 208. This indicates the critical role of ILC2s in the progression of liver diseases. The enrichment of ILC2s in human HCC has been associated with a higher recurrence and shorter survival rates. The reduction of e-cadherin in tumor cells leads to loss of killer cell lectin-like receptor G1 (KLRG1) expression in ILC2s, and this KLRG1- ILC2s subgroup secretes more CXCL2 recruitment neutrophils to form an immunosuppressive microenvironment 121. This suggests that targeting ILC2s could be a strategic approach to combat HCC. Furthermore, B cells participate in the ILC2s reprogramming, and the derived ICOSL signal induces IL-13 secretion *via* ICOS^+^ ILC2s, which promotes the progression of hepatitis and liver cancer 209. Recently, ILC3s have also been implicated in liver fibrosis as they enhance liver fibrosis by producing IL-22 and IL-17A when co-cultured with human HSC *in vitro*, and their secretion of IL-22 can block the anti-fibrotic effect of IFN-γ 13. In addition, high levels of IL-23 can help NCR^-^ ILC3s to secrete IL-17, suggesting poor clinical prognosis of HCC and weakened CD8+ T cell immunity 60.

Overall, ILC and cytokines should be focused in the early stage of the disease to shape its characteristic phenotype. This approach could be a more effective intervention for managing and treating liver diseases.

## Discussion

### ILC subpopulations are significant potential targets for cancer immunotherapy

ILC have an innate ability to communicate with cytokines and surrounding cells. This not only associated them with external stimuli and homeostasis but also with inflammation and tumor inevitably, given the complex and interconnected inflammation and tumor microenvironment. This dynamic interaction is crucial for understanding the role of ILC in health and disease. However, this communication ability is a two-way street. ILC not only kill pathogens, recruit and polarize immune cells, influence epithelial metaplasia, and even directly act on tumor stem cells, but are also shaped as they shape the immune microenvironment they house. This reciprocal influence is key to their functionality. For example, after the influence of IL-15 and TGF-β, NK transforms into ieILC1s, which inhibits cancer, and ILC1s (which has weak cytotoxicity) or anoxic environment mediates ILC2s into ILCreg phenotype, which promotes pancreatic cancer. Therefore, strategic modulation of ILC phenotypes could be a significant focus in cancer research.

### Key players in the progression of inflammation and cancer in the digestive System

Inflammation should be exclusionary and reparative; however, chronic inflammatory responses are detrimental to tumor control. Chronic inflammation can influence tumor development, distant metastasis, and resistance to therapy at the genetic level, through cellular interactions, and especially through the construction of the immune microenvironment. The spread of chronic inflammation to cancer is thought to be a relatively reversible and progressive process. The digestive system is a mucosal immune system that has extensive contact with the outside environment and comprises a large and complex immune microenvironment. Positive correlations have been demonstrated between HP-associated gastritis, NASH, chronic pancreatitis, intestinal inflammation, and cancers of various organs. These findings indicate that early intervention is crucial in these inflammatory processes to prevent cancer development. How to reverse or inhibit the progression of this process is crucial for early detection and treatment of cancer. Cytokine therapies involving ILC have provided additional options for immunotherapy in pancreatic and gastric cancers, such as dsNKG2D-IL-15, rIL-15, and L19-IL-2; however, ILC have higher efficiency and function. Further exploration into the diverse roles of ILC in cancer could identify new avenues for treatment.

ILC has been shown to pervade the digestive system and is associated with several key nodes from inflammation to cancer. First, ILC can inhibit the onset and progression of inflammation in the digestive system. This emphasizes the preventative role of ILC in disease progression. NK cells, synergistically with IL-15, can inhibit fibrosis in chronic pancreatitis by suppressing alveolar cell atrophy, preventing tissue collagen accumulation around blood vessels, and down-regulate pro-fibrotic genes. Furthermore, it has been observed that ILC2s produce IL-5 to mediate the IgA response in the early stages of HP infection, thereby eliminating pathogenic HP and thus inhibiting the development of gastritis. Moreover, androgens have been observed to prevent gastric inflammation and chemotaxis by blocking the production of proinflammatory cytokines of ILC2s. NK cells inhibit hepatitis progression and fibrosis by mediating apoptosis through the TRAIL/FasL pathway, granule cytotoxicity, and the production of IFN-γ, leading to HSC death. This highlights the therapeutic potential of leveraging ILC in inflammation control. However, as mentioned, the inflammatory environment also shapes or inhibits ILC function, and some ILC have opposite roles. Chronic HP infection inhibits NK cell toxicity, induces a long-term NK cell-associated IFN-γ response, and increases the risk of gastric cancer or inhibits IL-33, thereby limiting the activation of ILC2s that can clear it. When the fibrosis of the liver progressed to a later stage, HSC could inversely inhibit the anti-fibrotic capacity of NK cells *via* the TGF-β-dependent form. The specific role of different ILC in various stages of inflammation at key targets is crucial to prevent the progression of chronic digestive inflammation to tumors.

In addition to interacting with inflammation, ILC are involved in other pathways throughout digestive cancers. ILC can directly affect cancer cell proliferation and survival through their cytotoxicity, ADCC effects, and promotion of CSC differentiation. In addition, ILC can also affect signaling pathways such as IL-33/ST2, AREG-EGFR, AKT, STAT-3, *etc.* Moreover, ILC recruits, polarizes and functionally affects CD8^+^ T cells, Tregs, MDSC, neutrophils, and macrophages, which helps to shape the tumor immune microenvironment. ILC are also involved in angiogenesis and tumor migration. Almost all of these processes are associated with cytokines secreted by ILC.

### Current limitations and future directions

Although ILC have demonstrated considerable clinical potential, the majority of currently available information on ILC is derived from* in vitro* experiments and preclinical studies. Targeting ILC may have associated side effects and limitations; however, whether the results of preclinical studies are translated clinically and how these therapies can be personalized according to the tumor microenvironment or patient-specific factors should be studied. The practical translation of the above potential functions of ILC into clinically applicable immunotherapeutic regimens can be performed in the following ways. The first is to identify how to enhance the specific features of ILC per requirement. Cytokines, chemokines, microorganisms, and hormones offer many possibilities, which can be divided among different ILC groups. Another question is how to restore the suppressed function of ILC. This may require a more in-depth study of the specific mechanisms by which ILC interacts with surrounding immune cells, stromal cells, and microorganisms. Understanding these interactions is crucial for therapeutic development. Furthermore, when clarifying the pros and cons of ILC, it should be remembered that it performs different functions at different time points and can have ambiguous double-edged effects at the same time point. This complexity necessitates careful consideration in immunotherapy research. Therefore, the possible adverse effects of ILC should be considered when immunotherapy studies are conducted. This could, however, be solved by considering the plasticity of ILC. The role of ILC is inextricably linked to phenotype; therefore, more comprehensive studies of the typing and role of tissue-resident and peripheral blood ILC are needed. Further research in this area could lead to breakthroughs in targeted therapy. Finally, and most importantly, how ILC-related immunotherapies can be targeted should be assessed.

In addition to engineered cytokines, the genetic engineering of ILC is also worth exploring, which can be achieved *via* CAR-engineered NK (CAR-NK) cells. While optimizing manufacturing processes, scientists should also consider how to enhance CAR-NK cells' infiltration into solid tumors, cytotoxicity, and prolonged and reliable tumor surveillance. Moreover, CAR-NK cells have a better safety profile compared to engineered T cells. In the autologous setting, there is a reduced likelihood of cytokine release syndrome and neurotoxicity, while in the allogeneic setting, there is a reduced risk of graft-versus-host disease.

## Conclusion

In summary, ILC are valuable for tumor control as they influence inflammation in the digestive system and shape the immune microenvironment. Furthermore, they predominantly function by interacting with other cells and cytokines. Moreover, their high degree of plasticity and heterogeneity offers possibilities for the directional shaping of their relevant functions by influencing the environment in which they live. The construction of a comprehensive and detailed cytokine-ILC-immune microenvironment network is necessary for further understanding digestive disorders and to provide additional immunotherapeutic possibilities.

## Figures and Tables

**Figure 1:**
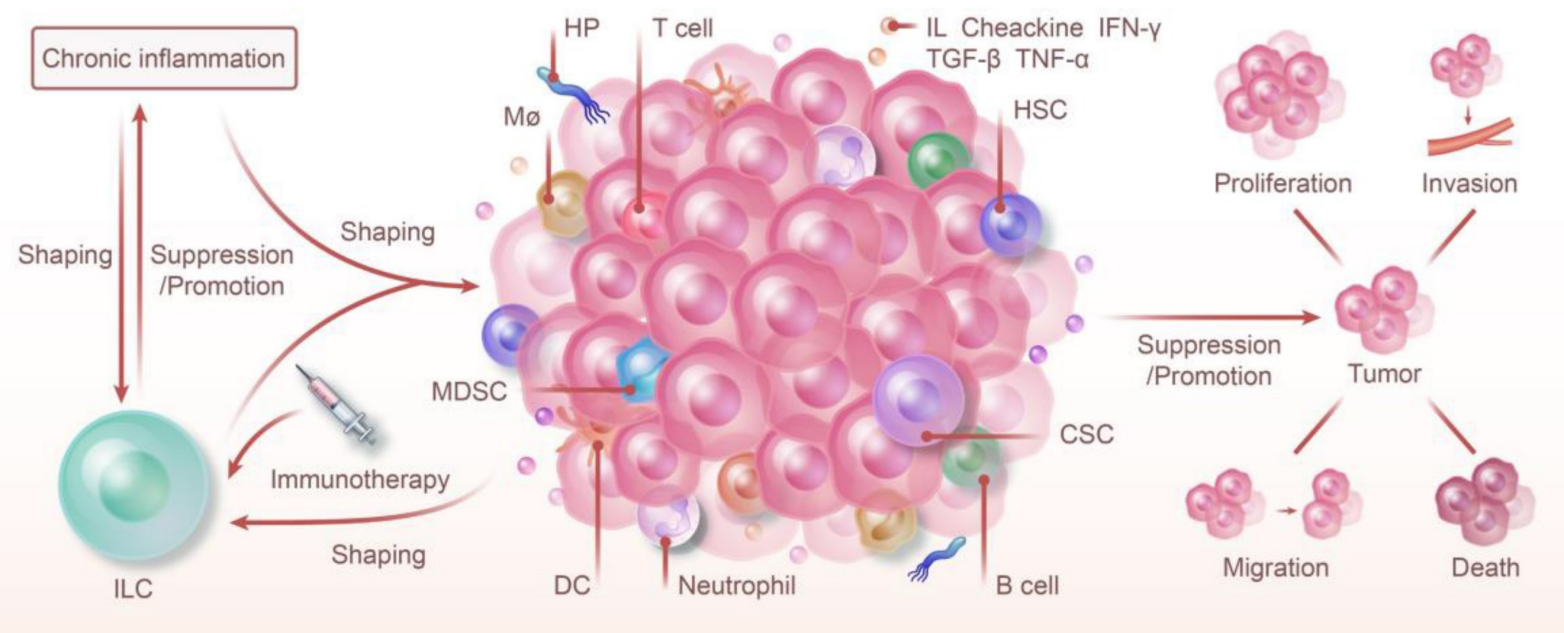
** ILC and Chronic Inflammation: Shaping Tumor Immunity and Therapy.** ILC and chronic inflammation synergistically shape the tumor immune microenvironment, jointly promoting or inhibiting the proliferation, invasion, metastasis, and death of tumors. The progression of chronic inflammation to cancer is regulated by ILC. Furthermore, chronic inflammation also modulates the types of ILC and influences their function. Because of their high degree of plasticity and unique function in various phenotypes, targeting ILC with immunotherapy offers a promising strategy for cancer prevention and control.

**Figure 2:**
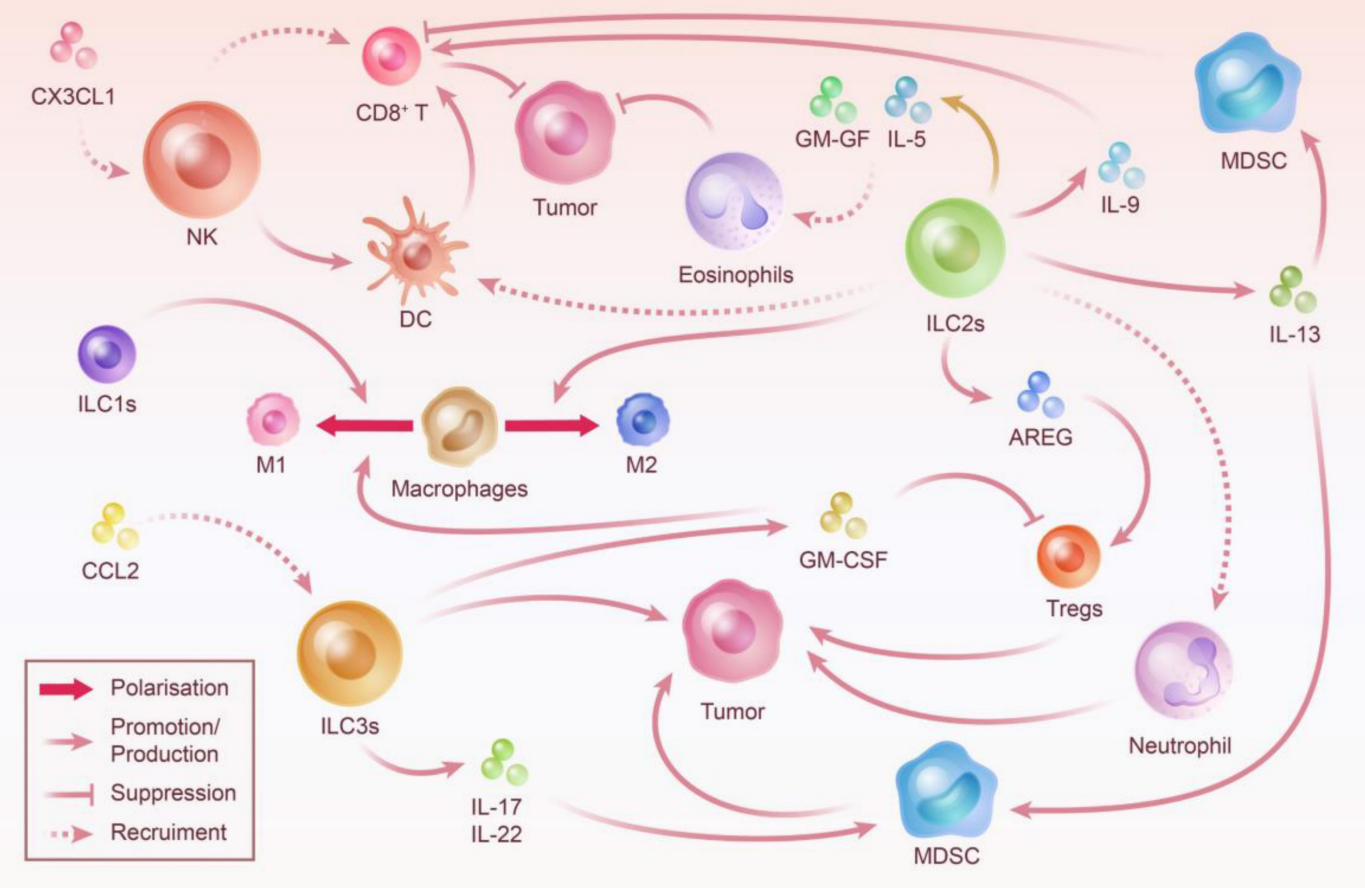
** Crosstalk Between ILC and Other Immune Cells: Steering Tumor Progression.** ILC prefer to be thoroughly integrated into the immune microenvironment in which they are embedded rather than working alone. The crosstalk between ILC and other immune cells is essentially linked with ILC-mediated tumor immune microenvironment construction. By recruiting immune cells, influencing their proliferation and activity, and polarizing neutral immune cells, ILC play an important role in controlling the direction of tumor progression.

**Figure 3:**
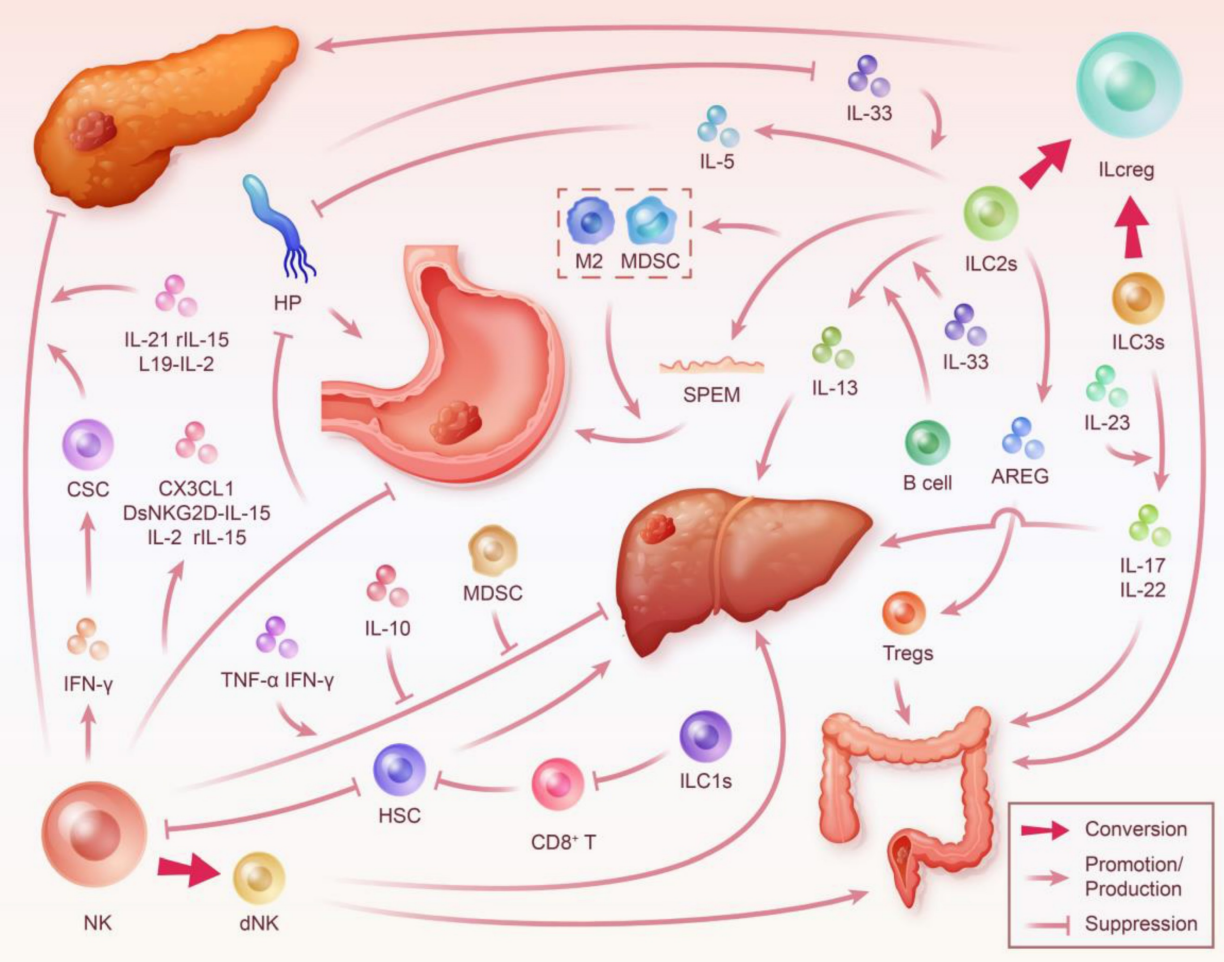
** ILC in the Digestive System Tumor Microenvironment.** During inflammation, different subsets of ILC interact with various factors including cytokines, immune cells, and tumor cells, significantly impacting the progression of digestive system tumors. This interaction highlights ILC's close relation to key targets critical for the transformation from digestive system inflammation to cancer, such as HSC and HP. ILC modulate the development and outcome of digestive system cancer through multiple pathways. However, it's important to note that the phenotype and function of ILC are also reciprocally shaped by the immune microenvironment. NK cells can inhibit the progression of chronic pancreatitis and PDAC by secreting IFN-γ to induce CSC differentiation, blocking fibrosis, promoting necrosis, and limiting proliferation in pancreatic cancer cells. Furthermore, they may also exacerbate gastritis and gastric carcinogenesis due to their weak cytotoxicity and mediation of a sustained IFN-γ response. This process is enhanced by HP and its secreted products, but targeted cytokine therapies may help NK cells regain their inhibitory capacity against gastric cancer. Moreover, NK cells promote hepatitis and fibrosis associated with HCC by inhibiting HSC. The conversion of NK cells to dNK mediates the progression of HCC and CRC. ILC1s may promote hepatic fibrosis by decreasing the killing of HSC by T cells. ILC2s inhibit the progression of gastritis to gastric cancer by killing HP while also mediating gastric cancer progression through SPEM and interaction with immunosuppressive cells. AREG secreted by ILC2s induces Tregs-mediated promotion of CRC. Transitioning from ILC2s to ILCreg promotes the progression of PDAC. ILC3s mediate colorectal inflammation by secreting IL-17 and IL-22 and contribute to cancer development. Their transition to ILCreg may also promote CRC.

**Table 1:** Effect of ILC on cancer.

Cancer	ILC subsets	Effects	Possible mechanism	Contributing factors	Inhibiting factors	References
Pancreatic cancer	NK	Suppression	NK cells induce CSC differentiation and inhibit pancreatic tumor growth and metastasis by secreting IFN-γ and TNF-α, ADCC, recruiting T cells, extensive necrosis and limited proliferation, and prevention of fibrosis.	IL-21, IL-15, IL-2		36, 120, 148, 149
Pancreatic cancer	ILC2s	Suppression	DC recruitment, CD8^+^T cell recruitment and activation, PD-1 pathway blocking enhancement	IL-33		74
Pancreatic cancer	ILC2s	Promotion	Transformation into ILCreg	Hypoxia, IL-33	AG	34, 73
Pancreatic cancer	ILC3s	Promotion	Production of IL-22 mediates AKT signaling.			153
CRC	NK	Promotion	Reduction of cytotoxicity, transformation to dNK, and promotion of angiogenesis	Tumor cells		164
CRC	ILC1s	Promotion	ILC1s drives intestinal epithelial and matrix remodeling.			165
CRC	ILC2s	Promotion	Production of AREG and enhancement of Tregs function, production of IL-13-mediated STAT6 activation, maintenance of tumor invasion MDSC	IL-25, IL-33	NMU	131, 167-169
CRC	ILC3s	Promotion	Production of IL-17, IL-22, and IFN-γ, transition to ILCreg, decreased communication with T cells	TGF-β		173-175
Gastric cancer	NK	Promotion	Persistent IFN-γ response, low cytotoxicity, and production of IFN-γ can contribute to gastric cancer cell invasive and metastatic capacity and reduce susceptibility to NK cells.	HP and its products, prostaglandin E2	IL-2,IL-15,CX3CL1	180, 182, 183, 185-190
Gastric cancer	ILC2s	Promotion	SPEM, dysregulation of Th1/Th2 responses together with increased MDSC and M2 phenotype macrophages.		Endogenous intragastric glucocorticoids, androgens	25, 140, 192
Gastric cancer	ILC2s	Suppression	ILC2s produce IL-5 and mediate IgA response to eradicate HP.	IL-33	HP	22, 111
HCC	NK	Suppression	NK cells promote HSC cell death and IFN-γ production and help macrophages polarize towards the M1 phenotype.	IFN-γ, TNF-α	HSC, IL-10, TGF-β, MDSC	23, 39, 109, 110, 138, 202, 203, 205
HCC	NK	Promotion	Transformation to dNK			207
HCC	ILC2s	Promotion	Secretion of IL-13, CXCL2 and recruitment of neutrophils	B cell		121, 208, 209
HCC	ILC3s	Promotion	Production of IL-22 and IL-17A, prevention of IFN-γ from antifibrosis, proliferation reduction, and increased apoptosis of CD8^+^T cells.			13, 60

## Abbreviations

ILC: Innate Lymphoid Cells
CRC: Colorectal Cancer
Hp: *Helicobacter pylori*
STAT-3: Signal Transducer and Activator of Transcription 3
IL-2: Interleukin-2
TNF: Tumor Necrosis Factor
TCR: T cell receptor
NK: Natural killer
ILC1s: Type 1 innate lymphoid cells
IFN: Interferon
AREG: Amphiregulin
NF-κB: Nuclear factor κB (NF-κB)
Th1: T helper type 1
TRAIL: Tumour necrosis factor-related apoptosis-inducing ligand
trNK: Tissue-resident NK
dNK: Decidual NK
ieILC1s: Intraepithelial ILC1s
TGF-β: Transforming growth factor-β
PDAC: Pancreatic ductal adenocarcinoma
PD-1: Programmed cell death protein-1
DC: Dendritic cells
MDSC: Myeloid-derived suppressor cells
KLRG1: Killer cell lectin-like receptor G1
CXCL2: C-X-C chemokine ligand 2
LTi: Lymphoid tissue inducer
AHR: Aryl hydrocarbon receptor
SCFA: Short-chain fatty acids
IBD: Inflammatory bowel disease
TLS: Tumor-associated tertiary lymphoid structures
NKT: Natural killer T
CCL21: Chemokine ligand 21
NASH: Non-alcoholic steatohepatitis
HCC: Hepatocellular carcinoma
HSC: Hepatic stellate cell
CSC: Cancer stem cell
ADCC: Antibody-dependent cell-mediated cytotoxicity
L19-IL2: L19-Interleukin-2
rIL-15: Recombinant IL-15
ILCreg: Regulatory innate lymphoid cells
AG: AG (nab-paclitaxel + gemcitabine)
NMU: Neurohormone U
rmIL-15: Recombinant mouse IL-15
SPEM: Spasmolytic polypeptide-expressing metaplasia
CAR-NK: CAR-engineered NK
Tregs: regulatory T cells
